# The prevalence of clinically relevant herb-drug interactions between herbal products and anti-cancer therapy in older adults with cancer – A cross-sectional study

**DOI:** 10.1016/j.rcsop.2025.100585

**Published:** 2025-03-17

**Authors:** Edwin J. Brokaar, Frederiek van den Bos, Johanneke E.A. Portielje, Loes E. Visser

**Affiliations:** a Department of Internal Medicine – Medical Oncology, University Medical Center Leiden, PO Box 9600, 2300 RC Leiden, the Netherlands; b Department of Pharmacy, Haga Teaching Hospital, PO Box 40551, 2504 LN The Hague, the Netherlands; c Department of Gerontology & Geriatrics, University Medical Center Leiden, PO Box 9600, 2300 RC Leiden, the Netherlands; d Department of Hospital Pharmacy, Erasmus University Medical Center, PO Box 2040, 3000 CA Rotterdam, the Netherlands; e Department of Epidemiology, Erasmus University Medical Center, PO Box 2040, 3000 CA Rotterdam, the Netherlands

**Keywords:** Herb-drug interaction, Oncology, Cancer treatment

## Abstract

**Background:**

The use of herbal supplements is highly prevalent amongst people with cancer and may lead to clinically relevant herb-drug interactions (HDIs) with their anti-cancer treatment. As the number of older adults with cancer increases, the numbers of older adults with cancer that are at risk of a HDI with anti-cancer treatment increases as well.

**Objective:**

The goal of this study was to establish the prevalence of potentially relevant HDIs in older adults who undergo anti-cancer treatment. Also, the overall use of herbal products in this population and possible associations with patient characteristics were investigated.

**Method:**

In this single center cross-sectional study patients aged ≥65 years were invited to participate if they underwent systemic anticancer treatment for a solid or hematological cancer. A questionnaire was developed to investigate the use of a selected set of frequently used herbs with known HDIs with regular medications. If a selected herb was used, the herb and oncological medication were assessed for potentially relevant HDIs. All potentially relevant HDIs were independently evaluated by two pharmacists on clinical relevance.

**Results:**

A total of 202 patients were included in the analysis. The mean age was 74 years and 54 % was male. The prevalence of potentially relevant HDIs was 4 % and overall herb use was 12 %. Thirteen potentially relevant HDIs were identified, of which 6 were judged to be clinically relevant. The clinically relevant HDIs concerned red yeast rice, red coneflower, turmeric, and cannabis. None of the patient characteristics were associated with overall herb use.

**Conclusion:**

Potentially relevant HDIs between herbal supplements and oncological treatment occur in 4 % of older adults with cancer and half of these are clinically relevant. Healthcare providers should question patients with cancer on the use of herbal supplements and monitor for relevant HDI with the treatment given.

## 1 Background

The use of complementary and alternative medicines (CAM) is widespread in patients with cancer, as 30–70 % uses CAM during cancer treatment ^1–31-3^, with herbal and dietary supplements being the most frequently used CAM.^4^ The proportion of cancer patients using herbal preparations varies from 19 to 70 %, with large differences between geographical locations or ethnic groups.^1^^,^^4^, ^5^, ^6^ Although patients make use of CAM for assumed health benefits, 40–70 % of patients do not inform their physicians about its use^7^^,^^8^ and the vast majority of herbal supplements is not registered in the medical or pharmaceutical records of the patients, as they are available as over-the-counter preparations or dietary supplements. As a result, healthcare providers frequently are unaware of the use of herbal supplements by cancer patients. Despite the fact that many patients believe herbal supplements are harmless as they are ‘natural’,^7^ herbal products may lead to clinically relevant herb-drug interactions (HDIs) with the anticancer treatment. HDIs are based on the same pharmacological principles as drug-drug interactions (DDIs) between regular drugs and frequently result from inhibition or induction of cytochrome P450 (CYP) enzymes or transporter proteins, thus increasing or decreasing exposure of the anticancer agent. HDIs and DDIs with anticancer agents may be highly relevant due to the narrow therapeutic window of anticancer medications and the massive impact of potential toxicity or treatment failure.^1^, ^2^, ^3^^,^^9^, ^10^, ^11^, ^12^ For example, St. John's Wort is known to induce CYP3A4 and may lead to undertreatment of the cancer when combined with CYP3A4-substrates imatinib or docetaxel.^3^

A recent literature review showed that 45 % of cancer patients who use herbal supplements, are at risk of HDIs.^13^ As the prevalence of cancer increases with age,^14^ the number of older cancer patients at risk for an HDI with anti-cancer medication increases as well.

In 2015, the Dutch Medicine Evaluation Board (MEB) launched an informational website to warn the general public about the risk of using herbal supplements in combination with regular medications, after research they conducted themselves that 10–17 % of the Dutch population uses herbal supplements.^15^ Part of this website is a section about ten commonly used herbs that are known to pose a risk of relevant HDIs when used in a pharmaceutical form, such as a decreased effect or increased toxicity of the regular drug. The information on these HDIs is based on the official drug product information and scientific literature. In this prospective cross-sectional study, the prevalence of potentially relevant herb-drug interactions (HDI) in older adults who undergo a treatment for cancer will be investigated. Furthermore, the overall use of herbal products in this population and possible associations with the patient characteristics sex, age, oncological specialism, number of regular systemic drugs, and level of education are explored.

## 2 Methods

### 2.1 Setting and population

This cross-sectional study was performed at the Haga Hospital in The Hague, the Netherlands. Patients were invited to participate if they were 65 years or older and underwent systemic anticancer treatment for a solid or hematological cancer. Patients were not invited if they received treatment with monoclonal antibodies exclusively, as these agents do not have any potential HDIs. Patients were also excluded if they did not speak Dutch or English. All patients received oral and written information about the study and provided a written consent. The Medical Ethical Committee (METC-ZWH) concluded that the Medical Research Involving Human Subjects Act (WMO) did not apply and no ethics approval was required. This study was performed after the subsequent approval of the management board of the Haga Hospital. Participants were recruited from May 2021 until November 2022.

### 2.2 Data collection

Polypharmacy, oncological specialism and oncological treatment were extracted from the electronic health records. Polypharmacy was defined as the use of ≥5 drugs that were used systemically or inhaled, taken continuously or as needed, and if it was not part of the cancer treatment or supportive cancer therapy, such as anti-emetics, granulocyte colony stimulating factor (G-CSF) or drugs for bone metastases. Drugs were counted based on ATC5-level and combination drugs were counted by their separate active substances.

For analytical purposes, age was grouped into the age groups 65–74 years and ≥ 75 years and level of education was grouped into lower level of education (none – highschool) and higher level of education (bachelor or higher).

### 2.3 Questionnaire

A questionnaire was completed by one of the researchers while interviewing the patient (preferred option) or by the patients themselves. Patients were interviewed face-to-face during a visit to the clinic or by telephone.

The questionnaire consisted of three parts, part A, B, and C. Part A were questions about herbal supplement use in general, part B inquired after the use of a selected set of herbal products, and part C was for registering other herbal supplements not mentioned in part B. A translated version of the HERb-Drug-Interactions with CHEMotherapy (HERDICHEM) questionnaire is provided in Supplement 1.

The specific set of herbal supplements selected for part B was based on the information from the Dutch MEB. This information is presented on the website of the MEB to raise awareness amongst the general population about ten frequently used herbs that have known HDIs with prescription drugs.^15^ Three other preparations from herbal origin (red yeast rice, cannabis, and Cimicifuga or Black cohosh) were added based on their frequent use amongst cancer patients and their HDI potential (expert opinion). The selected herbal supplements were preparations containing St John's wort, red yeast rice, American ginseng, red sage, turmeric, ginkgo, green tea, garlic, milk thistle, valerian, red coneflower, black cohosh and cannabis. Turmeric, green tea and garlic were only relevant when used as herbal supplements, not if used as beverage (green tea) or for cooking (garlic, turmeric).

### 2.4 HDI assessment

When a patient used a herbal supplement from part B, the research team screened for potential HDIs with their cancer treatment using the Interaction Checker from the website Natural Medicines.^16^ Every potential HDI was assessed for clinical relevance by two pharmacists independently. Disagreements on the clinical relevance were resolved by consensus and if consensus was not reached, the HDI was discussed with a third pharmacist for the final decision. If a potentially relevant HDI was identified, the patient and/or their oncologist were informed about the advice from the research team on that HDI as part of routine pharmaceutical care provided by the pharmacists of the hospital.

### 2.5 Outcomes

The primary outcome was the prevalence of potentially relevant HDI between the selected herbal supplements and the cancer treatment of the patient. Secondary outcomes were the overall prevalence of herbal supplement use, and patient's general knowledge and attitude towards herbal supplements. Furthermore, associations between age, sex, education level, number of drugs, polypharmacy, oncological specialism and the use of herbal supplements were studied. Ethnicity could not be recorded due to very strict regulations concerning such information in the Netherlands, despite ethnicity being a potentially confounding variable.

### 2.6 Analysis

The prevalence of potentially relevant HDI, overall use of herbal supplements and the results from Part A of the questionnaire were analyzed using descriptive statistics and expressed as a percentage of all patients. The associations between the overall use of herbal products and patient characteristics were analyzed using univariate logistic regression. A *p*-value <0.05 was considered statistically significant. All analyses were performed using IBM SPSS version 28.

## 3 Results

**Fig. 1:**
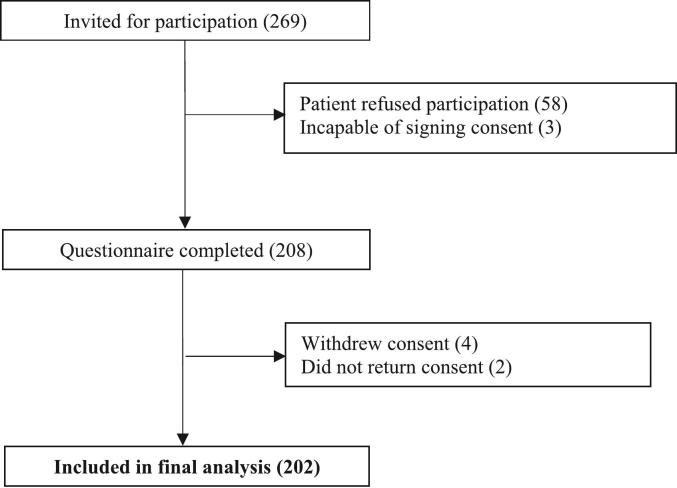
Recruitment and exclusion.

Two hundred sixty nine patients were invited to participate in this study, of which 58 refused participation and 3 were not able to sign the informed consent. Four patients withdrew their signed consent and 2 patients provided oral consent during the telephone interview, but did not return the signed consent. Therefore, a total of 202 patients were included in the analysis. The flowchart of recruitment and exclusion is shown in Fig. 1. The mean age was 73 years, 54 % was male and 67 % had polypharmacy. The level of education was low for 64 % and high for 36 %. Forty-eight percent of patients received treatment from a medical oncologist, 37 % from a hematologist and 15 % from a pulmonologist. All patient characteristics are presented in Table 1.

**Table 1:** Patient characteristics.

	ALL (*N* = 202)
Age, years (mean (95 %-CI))	73.1 (72.3–73.8)
65–75 years	122 (60.4 %)
75+ years	80 (39.6 %)

Sex	
Male	109 (54.0 %)
Female	93 (46.0 %)

Level of education^a^	
Low	130 (64.4 %)
High	72 (35.6 %)

Oncological specialism	
Medical oncology	97 (48.0 %)
Hematology	75 (37.1 %)
Pulmonology	30 (14.9 %)

Polypharmacy⁎	
Yes	136 (67.3 %)
No	66 (32.7 %)

Abbreviations: 95 % CI – 95 % confidence interval.

a Low = none-highschool; high = bachelor or higher.

⁎ ≥5 drugs.

Sixty-five percent of the interviews were taken face-to-face, 34 % by telephone and 1 % of questionnaires were filled in by the patient themselves.

### 3.1 Relevant HDI

Thirteen potentially relevant HDI were identified in 8 patients, which means a prevalence of 4 %. Six potentially relevant HDI were deemed to be clinically relevant and 7 were not. The 6 clinically relevant HDI occurred in 4 patients and concerned red yeast rice (1), red coneflower (2), turmeric (2) and cannabis CBD (1). For 5 clinically relevant HDI, the recommendation was to discontinue the herbal supplement and for 1 HDI, the recommendation was to monitor for potential toxicity of the herbal supplement. The 6 clinically relevant HDI are presented in Table 2 and the overview of all potential HDI are shown in Supplemental Table S3.

**Table 2:** Relevant HDIs.

Herbal supplement	Oncolytic drug	Description of HDI⁎	Recommendation
Red yeast rice	Olaparib	Inhibition of CYP3A4 by olaparib could lead to increased concentration and risk of toxicity lovastatin in red yeast rice.	Monitor toxicity of red yeast rice
Red coneflower	Olaparib	Red coneflower could induce hepatic CYP3A4 but inhibit intestinal CYP3A4. The Net effect may vary per CYP3A4 substrate but is uncertain/unpredictable olaparib.	Discontinue herbal supplement
Cannabis (CBD)	Paclitaxel	CBD might inhibit CYP2C8, leading to increased toxicity of paclitaxel.	Discontinue herbal supplement
Turmeric	Bortezomib	Antioxidative properties of turmeric could decrease the effect of bortezomib.	Discontinue herbal supplement
Turmeric	Melphalan	Antioxidative properties of turmeric could decrease the effect of melphalan.	Discontinue herbal supplement
Red coneflower	Cyclophosphamide	Red coneflower could induce hepatic CYP3A4 but inhibit intestinal CYP3A4. Net effect may vary per CYP3A4 substrate but is uncertain/unpredictable for cyclophosphamide.	Discontinue herbal supplement

Abbreviations: CYP – cytochrome P450 enzyme.

⁎ Source: Natural Medicines Interaction Checker^16^.

Of all respondents, 31 % were aware that herbal supplements can have an effect on the treatment of cancer and amongst patients who did use herbal supplements during cancer treatment, this was 58 %. The overall use of herbal supplements was 12 % and 13 patients (6 %) used 1 or more of the selected herbal supplement; 8 patients used one selected herbal supplement and 5 patients used 2 of the selected herbal supplements. Thus, a total of 18 of the selected herbal supplements were used during treatment for cancer, mainly red coneflower (*n* = 5) and cannabis (*n* = 4). Less frequently used were turmeric (*n* = 3), red yeast rice (*n* = 2), valerian (n = 2), ginkgo (*n* = 1), and garlic (n = 1). Supplemental Table S1 shows the frequency of use of each selected herb including the distribution of CBD and THC of the cannabis supplements. None of the patient characteristics were associated with the use of herbal supplements, see Table S2.

### 3.2 Patient's knowledge and beliefs

Out of 24 patients who used herbal supplements, 15 (63 %) indicated that they informed their oncologist about its use, for 7 patients (29 %) the oncologist was not aware of the herbal use, and 2 patients (8 %) did not know whether the oncologist was informed or not. Sixteen patients (67 %) stated that they would discontinue the herbal supplement if the oncologist recommended them to do so, 4 (17 %) were uncertain and 4 (17 %) would not. Sixty-seven percent of the patients using herbal supplements did so on their own initiative. Other reasons were recommendations from non-healthcare providers (13 %), alternative healthcare providers (25 %), or a cardiologist (4 %).

## 4 Discussion

The primary goal of this study was to investigate the prevalence of potentially relevant HDIs in older adults who were treated for cancer in a Dutch teaching Hospital. The results show a prevalence of 4 % in this population, 13 potentially relevant HDIs with selected herbal supplements occurred in 8 patients. Six HDIs in 4 patients were assessed to be clinically relevant and led to a recommendation about the herbal supplement. Furthermore, it was found that the overall use of herbal supplements was 12 % in older adults with cancer and nearly two-thirds of the users had informed their oncologist about the use of herbal supplements. Seventeen percent of the users would not discontinue the use of herbal supplements if their oncologist advised them to do so.

In this study, 8 out of 24 (33 %) users of herbal supplements were at risk for a potential HDI, but only in 4 patients an HDI was assessed to actually be clinically relevant. This is comparable to the results of a French study, who found that more than one-third of the users of herbal supplements had a potentially relevant HDI.^1^ In that study, however, the prevalence of potential HDIs in the entire population was 8 % compared to 4 % in our study. This may be explained by the fact that in the French study only patients with oral anticancer agents were included. Oral anticancer agents show a higher prevalence of drug-drug interactions and HDIs than anticancer agents overall.^17^ Lam et al. found in their review that 45 % of cancer patients with herbal supplement use are at risk of HDIs.^13^ A possible explanation for the lower percentage in our study is that we limited this study to a selection of the most frequently used herbs in the Dutch population with possible HDIs. Also, oncologists and oncology nurses in our hospital often inquire after the use of herbal supplements before the start of a new treatment and consult with a pharmacist about possible HDIs. As a result, potentially interacting combinations of herbal supplements with cancer treatment are frequently identified and eliminated before the start of the treatment. Furthermore, most oncologists in our clinic actively discourage the use of any herbal supplements in cancer patients, which also may explain the lower prevalence of their use in our population compared to other studies. The approach of active inquiry after the use of herbal supplements in cancer patients and pre-emptive screening for HDIs is not a common practice across Dutch hospitals. Therefore, it cannot be ruled out that the prevalence of use of herbal supplements and the prevalence of relevant HDIs is higher in a similar population in other Dutch hospitals. We recommend that medication reviews as part of patient intake ideally should include herbal drugs and healthcare professionals in oncology should actively inquire after the use of herbal supplements and discuss potential HDIs, risks and benefits with their patients.

Other investigators deemed 23/25 (92 %) of the potential HDIs not relevant and only 2/25 (8 %) relevant in a population of cancer patients in a university medical clinic.^18^ In this study, 6/13 (46 %) of the potential HDIs were assessed clinically relevant. The much larger proportion of clinically relevant HDIs in this study may be explained by the selection of herbs with known HDI-potential in our study. Furthermore, the assessment in this study of the potential relevance of HDIs and subsequent recommendations were quite cautious and the other investigators may have had a less hesitant approach.

The overall use of herbal supplements in our population (12 %) is comparable to that of the general Dutch population (10–17 %),^19^ but much lower than found in other studies amongst cancer patients (12 % to 70 %).^1^^,^^4^^,^^5^^,^^18^ A possible explanation is that oncologists and oncology nurses discourage the use of herbal supplements in general during cancer treatment in combination with the screening for HDIs in patients who do use herbal supplements. Also, a large variability in prevalence may be caused by varying definitions with respect to herbal supplements or herbal medicine.

In a primary care setting, 45 % of users of natural drugs – defined as products including herbs, animal parts and minerals in a pharmaceutical form – never informed their physician and 11 % did so only rarely.^7^ In that setting, the initiative for reporting the use lies with the patient primarily. Despite the active approach of the oncology department in our clinic, the percentage of patients not reporting the use of herbal supplement is only marginally lower (38 %). Another recent study on CAM use in a Swedish oncology clinic revealed that 35 % of cancer patients make use of CAM after the diagnosis of cancer and 70 % of these did not report CAM use to their oncologist. Herbal medicines and herbal tea accounted for only 9 % of all CAM use and the authors did not mention whether the various CAM modalities were reported differently to the oncologists.^8^ That study furthermore showed that female patients and patients with a university degree had higher use of CAM and also showed an association for lower CAM use with increasing age, but do not specify these findings for the various CAM modalities. This study did not show any associations for use of herbal supplement with these or other patient characteristics.

A total of 13 potential HDIs were identified, of which 6 were assessed clinically relevant and led to a recommendation. Assessing the relevance of a potential HDI in cancer therapy is not straightforward. For example, data on the use of substances with pronounced anti-oxidative properties may impair the effect of cytotoxic chemotherapy, but may also alleviate toxicity.^20^, ^21^, ^22^ Substances that increase oxidative stress may have both positive and negative properties with respect to cancer growth and therapy.^23^ Furthermore, many data on HDI are derived from in vitro research and the effect of the HDI on clinical endpoints is not established. Even more, constituents and pharmacologically active compounds in plant-based supplements or herbal medicines are not standardized and monitored as tightly as in regular medications, which leads to a larger possible variation in effect of the HDI. Turmeric may inhibit CYP3A4 and may affect exposure to CYP3A4 substrates such as imatinib and paclitaxel. Although bortezomib is not considered a sensitive CYP3A4 substrate, the World Health Organization (WHO) database of individual case safety reports mention a HDI between turmeric and bortezomib describing increased toxicity,.^24^ Also, turmeric exhibits antioxidant effects that may impair the cytotoxic effects of alkylating agents such as melphalan or cyclophosphamide. In vitro research suggests that antioxidative properties may be related to the dose applied.^25^ Cannabidiol (CBD) inhibits CYP2C19 and CYP2C9, but might also inhibit CYP3A4 and CYP2C8^26^ and may exhibit HDIs with e.g. cyclophosphamide of ruxolitinib. Although we only studied CBD when used in the form of a pharmaceutical preparation that is readily available in our country, many patients may smoke cannabis. Smoking cannabis also induces CYP1A2, thus affecting another range of medications that are not affected by CBD alone.^27^

A third difficulty in assessing the effect of an HDI is that a possible reduction in efficacy of the cancer treatment cannot be measured. Neo-adjuvant and adjuvant chemotherapy reduce the probability of cancer-related death in the future, and a modification of the effect of an HDI on that probability cannot be estimated even remotely. As a result of all these uncertainties, discontinuation of the herbal supplement was recommended even if only a theoretical possibility was present for a decreased efficacy of the cancer treatment.

### 4.1 Strengths and weaknesses

A strength of this study is that 99.5 % of all questionnaires were filled by a researcher during personal contact or a telephone call, which means that possible misunderstanding of the questions or definition of herbal supplement could be elucidated. A weakness may be that only a selected number of herbal supplements with possible HDI were selected. Although this selection probably covers the vast majority of occurring HDI, a higher yield in potentially relevant HDI could have been found if all herbal supplements were assessed. Furthermore, the effect of ethnicity on the use of herbal supplements could not be studied, as ethnicity could not be recorded. Although recall bias cannot be excluded completely, we believe it does not play a major role in this study. Patients were interviewed by a researcher about the current use of herbal supplements during the period they received anticancer therapy. The study was performed in a single hospital with an above average awareness of CAM use and with a relatively small sample size, which may have resulted in selection bias.

### 4.2 Future research

Ideally, future research should focus on the effect of potentially relevant HDI with oncological treatment on clinical endpoints, such as mortality, toxicity, or progression free survival. Due to the low prevalence of relevant HDIs and the large number of patients needed to prove an effect of a HDI on a clinical outcome with a certain oncological treatment, such a study is unlikely to be performed. Future research could assess the impact of herbal supplements on serum drug levels of oncological medications. Furthermore, as the percentage of patients taking herbal supplements and the nature of these supplements vary across countries or geographical regions, this type of research has to be repeated in other countries, and training and education methods for healthcare professionals should be explored on how to inquire after use of herbal supplements. An overview of all identified relevant HDIs as a guide would support the feasibility of such studies, as future research could focus on those herbal supplements primarily.

## 5 Conclusions

The use of herbal supplements in older adults with cancer is comparable to that of the general Dutch population. Potentially relevant HDIs between herbal supplements and oncological treatment occur in 4 % of older adults with cancer and half of these HDIs are clinically relevant. Healthcare providers should question patients with cancer on the use of herbal supplements and monitor for relevant HDI with the treatment given.

## CRediT authorship contribution statement

**Edwin J. Brokaar:** Writing – original draft, Project administration, Methodology, Formal analysis, Data curation, Conceptualization. **Frederiek van den Bos:** Writing – review & editing, Supervision, Conceptualization. **Johanneke E.A. Portielje:** Writing – review & editing, Supervision, Conceptualization. **Loes E. Visser:** Writing – review & editing, Supervision, Methodology, Conceptualization.

## Declaration of competing interest

The authors have nothing to declare.

## Appendix A Supplementary data

Supplementary material1 Supplementary tables Supplementary material1

Supplementary material2 HERDICHEM Questionnaire Supplementary material2

## References

[1] Clairet A.L., Boiteux-Jurain M., Curtit E. (2019). Interaction between phytotherapy and oral anticancer agents: prospective study and literature review. Med Oncol (Northwood, London, England).

[2] Collado-Borrell R., Escudero-Vilaplana V., Romero-Jiménez R., Iglesias-Peinado I., Herranz-Alonso A., Sanjurjo-Sáez M. (2016). Oral antineoplastic agent interactions with medicinal plants and food: an issue to take into account. J Cancer Res Clin Oncol.

[3] Goey A.K., Beijnen J.H., Schellens J.H. (2014). Herb-drug interactions in oncology. Clin Pharmacol Ther.

[4] Sparreboom A., Cox M.C., Acharya M.R., Figg W.D. (2004). Herbal remedies in the United States: potential adverse interactions with anticancer agents. J Clin Oncol.

[5] Alsanad S.M., Howard R.L., Williamson E.M. (2016). An assessment of the impact of herb-drug combinations used by cancer patients. BMC Complement Altern Med.

[6] Arcury T.A., Grzywacz J.G., Bell R.A., Neiberg R.H., Lang W., Quandt S.A. (2007). Herbal remedy use as health self-management among older adults. J Gerontol B Psychol Sci Soc Sci.

[7] Giveon S.M., Liberman N., Klang S., Kahan E. (2004). Are people who use “natural drugs” aware of their potentially harmful side effects and reporting to family physician?. Patient Educ Couns.

[8] Kallman M., Bergstrom S., Carlsson T. (2023). Use of CAM among cancer patients : results of a regional survey in Sweden. BMC Complement Med Ther.

[9] Cheng Y.Y., Hsieh C.H., Tsai T.H. (2018). Concurrent administration of anticancer chemotherapy drug and herbal medicine on the perspective of pharmacokinetics. J Food Drug Anal.

[10] Haefeli W.E., Carls A. (2014). Drug interactions with phytotherapeutics in oncology. Expert Opin Drug Metab Toxicol.

[11] Hu Z., Yang X., Ho P.C. (2005). Herb-drug interactions: a literature review. Drugs.

[12] Thomas-Schoemann A., Blanchet B., Bardin C. (2014). Drug interactions with solid tumour-targeted therapies. Crit Rev Oncol Hematol.

[13] Lam C.S., Koon H.K., Ma C.T. (2022). Real-world data on herb-drug interactions in oncology: a scoping review of pharmacoepidemiological studies. Phytomedicine.

[14] Siegel R.L., Miller K.D., Jemal A. (2017). Cancer statistics, 2017. CA Cancer J Clin.

[15] Board CtBvGME Patient information about herbs. https://www.cbg-meb.nl/onderwerpen/medicijninformatie-kruiden.

[16] Center TR. Natural medicines interaction checker. Therapeutic Research Center (https://naturalmedicines.therapeuticresearch.com/).

[17] van Leeuwen R.W., van Gelder T., Mathijssen R.H., Jansman F.G. (2014). Drug-drug interactions with tyrosine-kinase inhibitors: a clinical perspective. Lancet Oncol.

[18] Jermini M., Dubois J., Rodondi P.Y. (2019). Complementary medicine use during cancer treatment and potential herb-drug interactions from a cross-sectional study in an academic Centre. Sci Rep.

[19] Jeurissen S.M.F., Buurma-Rethans E.J.M., Beukers M.H., Jansen-van der Vliet M., van Rossum C.T.M., Sprong R.C. (2018). Consumption of plant food supplements in the Netherlands. Food Funct.

[20] Fernando W., Rupasinghe H.P.V., Hoskin D.W. (2019). Dietary phytochemicals with anti-oxidant and pro-oxidant activities: a double-edged sword in relation to adjuvant chemotherapy and radiotherapy?. Cancer Lett.

[21] Khurana R.K., Jain A., Jain A., Sharma T., Singh B., Kesharwani P. (2018). Administration of antioxidants in cancer: debate of the decade. Drug Discov Today.

[22] van Gorkom G.N.Y., Lookermans E.L., Van Elssen C., Bos G.M.J. (2019). The effect of vitamin C (ascorbic acid) in the treatment of patients with cancer: a systematic review. Nutrients.

[23] Marengo B., Nitti M., Furfaro A.L. (2016). Redox homeostasis and cellular antioxidant systems: crucial players in cancer growth and therapy. Oxidative Med Cell Longev.

[24] Pochet S., Lechon A.S., Lescrainier C. (2022). Herb-anticancer drug interactions in real life based on VigiBase, the WHO global database. Sci Rep.

[25] Somasundaram S., Edmund N.A., Moore D.T., Small G.W., Shi Y.Y., Orlowski R.Z. (2002). Dietary curcumin inhibits chemotherapy-induced apoptosis in models of human breast cancer. Cancer Res.

[26] Bansal S., Paine M.F., Unadkat J.D. (2022). Comprehensive predictions of cytochrome P450 (P450)-mediated in vivo cannabinoid-drug interactions based on reversible and time-dependent P450 inhibition in human liver Microsomes. Drug Metab Dispos.

[27] Anderson G.D., Chan L.N. (2016). Pharmacokinetic drug interactions with tobacco, cannabinoids and smoking cessation products. Clin Pharmacokinet.

