# Supplementary material for: The prevalence of clinically relevant herb-drug interactions between herbal products and anti-cancer therapy in older adults with cancer – A cross-sectional study
Source: Explor Res Clin Soc Pharm. 2025 Mar 17;18:100585. doi: 10.1016/j.rcsop.2025.100585 (PMC11982495; doi:10.1016/j.rcsop.2025.100585)
Supplement: Supplementary material1 — Supplementary tables [file mmc1.docx]

**SUPPLEMENTARY TABLES**

**Table S1. Number of patients using selected herbs**

| **Herbal supplement** | **Number of patients** |
| --- | --- |
| Red coneflower | 5 |
| Cannabis | 4 |
| *Only or primarily CBD* | *1* |
| *Only or primarily THC* | *1* |
| *THC + CBD* | *2* |
| *Unknown* | *0* |
| Turmeric | 3 |
| Red yeast rice | 2 |
| Valerian | 2 |
| Ginkgo | 1 |
| Garlic | 1 |
| Milk thistle | 0 |
| Black cohosh | 0 |
| St John’s wort | 0 |
| American Ginseng | 0 |
| Red sage | 0 |
| Green tea | 0 |

**Table S2. Analysis of patient characteristics and overall herbal supplement use**

|  | **Herb use (%)** | **Odd ratio (95% CI)** | **p-value** |
| --- | --- | --- | --- |
| **All** | 11.9 |  |  |
| **Age** |  |  | 0.822 |
| 65-74 years | 12.3 | 1.00 (ref) |  |
| 75+ years | 11.3 | 0.90 (0.38 – 2.18) |  |
| **Sex** |  |  | 0.679 |
| Female | 12.9 | 1.00 (ref) |  |
| Male | 11.0 | 0.84 (0.36 – 1.96) |  |
| **Level of education** |  |  | 0.270 |
| Lower | 10.0 | 1.00 (ref) |  |
| Higher | 15.3 | 1.62 (0.69 – 3.84) |  |
| **Oncological specialism** |  |  | 0.632 |
| Internal medicine | 12.4 | 1.00 (ref) |  |
| Hematology | 13.3 | 1.09 (0.44 – 2.68) |  |
| Pulmonology | 6.7 | 0.51 (0.11 – 2.40) |  |
| **Polypharmacy (≥5 drugs)** |  |  | 0.148 |
| No | 16.7 | 1.00 (ref) |  |
| Yes | 9.6 | 0.53 (0.22 – 1.25) |  |

**Table S3. Overview of assessment of potentially relevant HDIs**

| **Herbal supplement** | **Oncolytic** | **Clinically relevant** | **Recommendation** |
| --- | --- | --- | --- |
| Red yeast rice | Olaparib | Yes | Monitor toxicity of red yeast rice |
| Red coneflower | Olaparib | Yes | Discontinue herbal supplement |
| Cannabis (CBD) | Paclitaxel | Yes | Discontinue herbal supplement |
| Turmeric | Bortezomib | Yes | Discontinue herbal supplement |
| Turmeric | Melphalan | Yes | Discontinue herbal supplement |
| Red coneflower | Cyclophosphamide | Yes | Discontinue herbal supplement |
| Red coneflower | Paclitaxel | No | n/a |
| Turmeric | Paclitaxel | No | n/a |
| Red coneflower | Enzalutamide | No | n/a |
| Garlic | Enzalutamide | No | n/a |
| Valerian | Paclitaxel | No | n/a |
| Red coneflower | Paclitaxel | No | n/a |
| Valerian | Etoposide | No | n/a |

Abbreviations: n/a – not applicable
