# Supplementary material for: The prevalence of clinically relevant herb-drug interactions between herbal products and anti-cancer therapy in older adults with cancer – A cross-sectional study
Source: Explor Res Clin Soc Pharm. 2025 Mar 17;18:100585. doi: 10.1016/j.rcsop.2025.100585 (PMC11982495; doi:10.1016/j.rcsop.2025.100585)
Supplement: Supplementary material2 — HERDICHEM Questionnaire [file mmc2.pdf]

HagaZiekenhuis van Den Haag

HERDICHEM Questionnaire

Questionnaire to the HERDICHEM-study for researching the interaction between herbal supplements and chemotherapy.

Demographics of participant

Sex male / female

Age ……………. years
Highest level of education
- ☐ None
- ☐ Elementary school
- ☐ Middle school
- ☐ High school
- ☐ Bachelor
- ☐ Master
- ☐ PhD

To be completed by the researcher
ID
Questionnaire FTF / self / videocall / telephone
Completed by:
Date:
Initials:

Part A
General questions on the use of herbal supplements

1. I knew that some herbal supplements can affect cancer treatmen.
   - ☐ Yes
   - ☐ No

2. I am using herbal supplements during my treatment for cancer.
   - ☐ Yes → continue
   - ☐ No → you finished the questionnaire

3. My oncologist knows I am using herbal supplements.
   - ☐ Yes
   - ☐ No
   - ☐ I do not know

4. I would stop using herbal supplements if my oncologist would tell me to.
   - ☐ Yes
   - ☐ No
   - ☐ I do not know (yet)

5. I am using herbal supplements
   - ☐ On my own initiative
   - ☐ On advice of others such as family members, acquaintances, friends, neighbors
   - ☐ On advice of an alternative healthcare provider
   - ☐ Other reason, namely ………………………………………………………………………………………….

Part B

Check if you are using one or more of the following herbal supplements.

- ☐ St John's wort (Hypericum perforatum)
- ☐ Red yeast rice
- ☐ American Ginseng (Panax quinquefolius)
- ☐ Danshen or red sage (Salvia miltiorrhiza)
- ☐ Turmeric* (Curcuma longa)
- ☐ Cannabis. If yes, with which active substances:
    - ○ Only or primarily CBD
    - ○ Only or primarily THC
    - ○ THC + CBD
    - ○ Unknown
- ☐ Ginkgo (Ginkgo biloba)
- ☐ Green tea* (Camellia sinensis)
- ☐ Garlic* (Allium sativum)
- ☐ Milk thistle (Silybum marianum)
- ☐ Valerian (Valeriana officinalis)
- ☐ Red coneflower (Echinacea)
- ☐ Black cohosh (Cimicifuga)

☐ I do not use any of these herbal supplements

*Turmeric, green tea and garlic only when in use as herbal supplements. Do not check if green tea is used as beverage and do not check turmeric or garlic when used for cooking.

Part C

Write any other herbal supplements you are using in the box below.

Thank you very much for completing this questionnaire!

The research team.
